# Successful integration of HIV pre-exposure prophylaxis into a community-based HIV prevention program for female sex workers in Kolkata, India

**DOI:** 10.1177/0956462420983992

**Published:** 2021-02-18

**Authors:** Smarajit Jana, Protim Ray, Soma Roy, Abhijit Kadam, Raman R Gangakhedkar, B B Rewari, Stephen Moses, Marissa L Becker

**Affiliations:** 1 Durbar Mahila Samanwaya Committee, Kolkata, India; 2 National AIDS Research Institute, Pune, India; 3 28604 Indian Council of Medical Research, New Delhi, India; 4 WHO-SEARO, New Delhi, India; 5 Department of Community Health Sciences, Institute for Global Public Health, 8664 University of Manitoba, Winnipeg, Canada

**Keywords:** Pre-exposure prophylaxis, female sex workers, HIV, India

## Abstract

We assessed the impact of pre-exposure prophylaxis (PrEP) in the context of a community-based HIV program among female sex workers (FSWs) in Kolkata, India. This was an open-label, uncontrolled demonstration trial. HIV seronegative FSWs over 18 years were eligible. Participants were administered daily tenofovir/emtricitabine (TDF-FTC) with follow-up visits at months 1, 3, 6, 9, 12, and 15. Drug adherence was monitored by self-report, and a random subset of participants underwent plasma TDF testing. 843 women were screened and 678 enrolled and started on PrEP. Seventy-nine women (11%) did not complete all scheduled visits: four women died of reasons unrelated to PrEP and 75 withdrew, for a 15-month retention rate of 89%. Self-reported daily adherence was over 70%. Among those tested for TDF, the percentage of women whose level reached ≥40 ng/mL was 65% by their final visit. There were no HIV seroconversions, and no evidence of significant changes in sexual behavior. This study demonstrated the feasibility and effectiveness of PrEP for FSWs in Kolkata, with very high levels of adherence to PrEP and no HIV seroconversions. The integration of PrEP into an existing community-based HIV prevention program ensured community support and facilitated adherence.

## Introduction

India has the third largest HIV epidemic in the world, with the highest rates of HIV infection concentrated among key affected populations, including female sex workers (FSWs).^1^ In India, FSWs are at high risk of HIV through unprotected sex from both sex work partners and intimate partners.^2^ Condom use prevents HIV and other sexually transmitted infections (STIs), but not all FSWs are able to use condoms consistently and correctly, particularly with regular partners.^3^ In India, it is estimated that at least 20% of FSWs remain at high risk of acquiring HIV infection for this reason, despite the best efforts of HIV prevention programs.^4,5^ Other approaches are therefore needed to close this significant prevention gap.

Randomized clinical trials (RCTs) have demonstrated the efficacy of daily, oral tenofovir/emtricitabine (TDF-FTC) in preventing HIV infections among high-risk populations, including FSWs, in a wide range of settings.^6–10^ In 2012, the U.S. Food and Drug Administration approved the use of co-formulated TDF-FTC for HIV pre-exposure prophylaxis (PrEP) among adults at high risk of HIV infection. Pre-exposure prophylaxis has since been integrated into the national guidelines of more than 30 countries.^11^ However, important questions remain as to how to implement PrEP programs in “real-world” settings, including what populations would benefit most, how to optimally deliver PrEP and support adherence, and what potential impact PrEP might have on existing HIV prevention efforts.^12^ The World Health Organization has called for demonstration projects to examine the effectiveness of PrEP outside of clinical trials. However, only one study to date has evaluated the use of oral PrEP by FSWs or other high-risk populations in India.^13^

In this study, we present the results of a demonstration trial in Kolkata, India, designed to assess the feasibility and impact of delivering PrEP, integrated within an existing community-based HIV prevention program for FSWs in Kolkata, the capital of West Bengal, and one of the largest cities in India (population 15 million). We assessed whether daily oral PrEP could be added in a safe and effective manner to a package of HIV preventive interventions for FSWs. Specifically, we assessed whether sustained uptake and adherence to oral PrEP could be achieved, and we evaluated risks including any negative impact on perceptions of risk and on HIV prevention practices.

## Methods

### Design

This trial was an open-label, single-arm (uncontrolled) demonstration trial. The trial (registered on Clinicaltrials.gov, No. NCT02148094) was approved by Institutional Review Boards of the *Durbar Mahila Samanwaya Committee* (DMSC) and the University of Manitoba (Canada), and by the Indian Council of Medical Research, through the Health Ministry’s Screening Committee. The project followed all guidelines for research on human subjects as mandated by the Government of India.

### Population and settings

Kolkata has a concentrated HIV epidemic, with an estimated HIV prevalence of 2.2% among FSWs in 2015^14^, as compared to an HIV prevalence in the general adult population of 0.21%.^14^ This PrEP demonstration trial was conducted by the DMSC, a large community-owned sex work program comprising over 60,000 FSW members, who are largely brothel based.^15,16^ *Durbar Mahila Samanwaya Committee* has been providing focused HIV prevention and care for FSWs through peer education, HIV testing and counseling, condom promotion, and STI screening and treatment for almost 30 years. In preparation for this project, FSWs within the community were engaged in a series of consultations, where information on PrEP was provided, and questions and concerns were discussed. A small pilot study was conducted using both questionnaires and focus group discussions to assess the feasibility and acceptance of PrEP among FSWs.^5^ The results of this study, along with WHO guidelines, informed the design and implementation of the present trial.

### Eligibility

For the purposes of this study, sex work was defined as sex in exchange for money, wherein the price was negotiated prior to the sex event. Women were eligible to participate if they self-identified as currently active FSWs (paid for sex in the last 3 months), were 18 years or older, lived within the catchment area of DMSC and had no plans to relocate during the study time period, and were interested and willing to take PrEP. Women were excluded if they were already taking PrEP, were pregnant, or living with HIV or hepatitis B; or if they had evidence of abnormal renal function (creatinine clearance <60 mL/min), abnormal liver function, or other severe illnesses. The target sample size for this study was 600 participants.

### Study interventions and procedures

Female sex workers were recruited during routine outreach visits by DMSC peer educators (current or former FSWs who were trained as health workers). After providing information on potential benefits and adverse effects of PrEP, interested women were referred to DMSC-run clinics for screening and enrollment. Enrollment started in January 2016 and ended in October 2016. Final follow-up visits were completed in February 2018.

**Figure 1.**
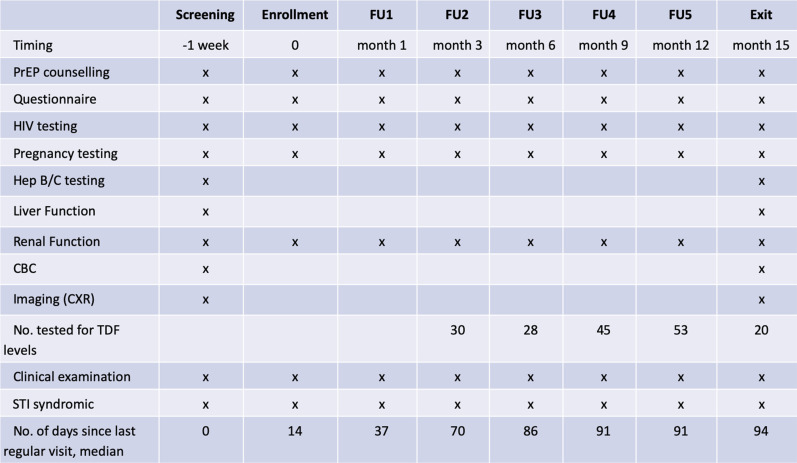
Trial schedule and procedures.

At the screening visit, the risks and benefits of PrEP were explained, and written informed consent was obtained prior to completing enrollment procedures (Figure 1). Pregnancy testing, HIV testing, and counseling were performed. HIV testing was conducted as per Indian national guidelines. Individuals testing positive for HIV were referred for HIV care. Further medical assessment included screening for hepatitis B and C, syphilis, gonorrhea, and chlamydia; complete blood count (CBC); liver and renal function testing; and imaging (chest X-ray and abdominal ultrasound). Treatment and/or referral for care were provided as indicated.

One week after screening, participants returned for their enrollment visit. They underwent repeat pregnancy and HIV testing (to confirm HIV negative status). Those who remained eligible were enrolled into the trial. They received HIV risk reduction counseling and in-depth counseling about PrEP, including assessment of potential barriers to adherence. The primary intervention was a once daily oral TDF-FTC (200 mg/300 mg) combination pill (Mylan Pharmaceuticals Pvt. Ltd., Taloja, Dist-Raigad, India). Participants were given a choice between two PrEP delivery options: weekly clinic pickup or home delivery by a peer educator every other day. Assessment and support for adherence, management of adverse events, and support for condom use were provided during scheduled clinic visits or by peer educators for those who chose home delivery. In addition, all participants were provided with standard HIV prevention services, including condoms, in accordance with Indian national guidelines.^17^

Clinic follow-up visits were scheduled at months 1, 3, 6, 9, 12, and 15 following enrollment (Figure 1). At each visit, information on adherence, adverse events, sexual risk factors, and experience using PrEP was gathered by a trained interviewer using a standardized questionnaire. In addition to a clinical examination (including STI syndromic screening performed by the attending physician), laboratory testing for HIV, pregnancy, and creatinine levels were also performed. At the final visit (15 months), testing was done for hepatitis B and C and other STIs, as well as CBC, liver, and renal function tests. A chest X-ray and abdominal ultrasound were also performed, similar to the enrollment visit. In addition, blood specimens were collected from over 10% of randomly selected participants between visits 3 and 15 and sent to the National AIDS Research Institute in Pune, India, for plasma TDF level testing. Tenofovir testing was conducted on plasma using liquid chromatography–mass spectrometry (SCIEX 4000 QTrap, along with the Shimadzu LC 20AD LC system).

### Statistical analysis

Information on eligibility, scheduled visits, drug dispensation, and adverse events were entered into a custom-made computer application. Information from questionnaires was entered into an EpiData database.^18^ Retention was defined by examining the number of scheduled clinical visits completed. Pre-exposure prophylaxis adherence was measured as the percentage of PrEP initiators who continued to use PrEP at each scheduled visit, including the final visit. Information on adherence was collected using questionnaires completed at each scheduled visit and from records maintained by peer educators. We report the proportion of participants at each visit who indicated that they had taken seven pills in the last week, as well as the proportion who reported having taken ≥4 pills in the last week.^19,20^

Detectable TDF levels were defined as an individual having a TDF level >0.31 ng/mL.^21,22^ We also used a cutoff level of ≥10 ng/mL, indicative of a pill taken in the last 24–48 h,^23^ and a cutoff level of ≥40 ng/mL believed to be a surrogate of clinical effectiveness.^22^

Occurrence of adverse events and information on sexual behavior (e.g., frequency and patterns of sexual activity and condom use) were collected using questionnaires completed at each scheduled visit, as well as by peer educators, using a pictorial tool developed for this purpose. Finally, the use of existing HIV prevention and related services was monitored. In addition to program monitoring data (e.g., the number of providers and peer educators trained in PrEP delivery, and number of sites prepared to distribute PrEP), logs were kept by peer educators to record their activities, such as the number of client visits made and PrEP doses dispensed.

## Results

Of 843 women screened, 115 (13.6%) were not eligible for enrollment in the study, mainly because of abnormal liver function (*N* = 66) and other illnesses (Table 1). Others excluded were 13 (1.5%) who tested positive for HIV and another 9 (1.1%) who tested positive for hepatitis B. After screening, 42 women (5.0%) did not return for enrollment, and another 8 (0.9%) declined to participate in the study. The remaining 678 women, representing 80.4% of all those screened, were enrolled and started on PrEP. Of these, 353 chose home delivery of medications and 335 chose clinic pickup.

**Table 1.** Enrollment and attrition.

	No.	%
Screened	843	
Pregnancy	13	1.5
HIV-positive	13	1.5
Hepatitis B-positive	9	1.1
Abnormal liver function	66	7.8
Kidney disease^a^	9	1.1
Other illness	17	2.0
Total ineligible^b^	115	13.6
Total eligible	728	86.4
Refused	8	0.9
Did not return	42	5.0
Enrolled	678	80.4
Died	4	0.5
Withdrew	75	8.9
Finished	599	71.1

^a^Kidney disease was defined as creatinine clearance ≥60 mL/min.

^b^This number is smaller than the sum of all eligibility reasons because some women had >1 reason for ineligibility.

### Baseline characteristics

The median age at enrollment was 28 years (Q1–Q3, 25–35) (Table 2). About 42% of enrolled women were illiterate, and only a few (7%) reported completing high school. Most women (64%) were widowed, divorced, or separated. About 23% reported using a non-barrier birth control method and another 31% had had a tubal ligation. Consistent condom use with occasional clients was reported by 91% of participants, but a smaller percentage (76%) reported using condoms consistently with repeat clients and only 10% with their intimate partners. Over 87% had been tested for HIV in the previous year. Regular alcohol consumption before sex work was reported by over 22% of women. Very few (<2%) reported illicit drug use. On average, those who chose to pick up their drug from clinics had fewer occasional clients, but were less likely to use condoms consistently, and more likely to have been tested or treated for STIs in the past 3 months.

**Table 2.** Baseline characteristics by the PrEP delivery method.

	Home delivery	Clinic pick-up	Total
Total, No.	353	325	678
Sociodemographics			
Age (years), median (Q1–Q3)	28 (25–35)	28 (25–35)	28 (25–35)
Education, n (%)			
Illiterate	155 (43.9)	128 (39.4)	283 (41.7)
Some school	166 (47.0)	181 (55.7)	347 (51.2)
High school or higher	32 (9.1)	16 (4.9)	48 (7.1)
Marital status, n (%)			
Never married	16 (4.5)	26 (8.0)	42 (6.2)
Married	98 (27.8)	101 (31.1)	199 (29.4)
Widow, divorced, or separated	237 (67.1)	198 (60.9)	435 (64.2)
Unknown	2 (0.6)	0 (0.0)	2 (0.3)
Income last month, 1000s rupees, median (Q1–Q3)	14 (8–25)	10 (6–20)	12 (7–22)
Reproductive health			
No. of living children, median (Q1–Q3)	1 (1–2)	1 (1–2)	1 (1–2)
Using a non-barrier birth control method, n (%)	81 (22.9)	75 (23.1)	156 (23.0)
Had tubal ligation, n (%)	113 (32.0)	94 (28.9)	207 (30.5)
Planning to become pregnant, n (%)	21 (5.9)	38 (11.7)	59 (8.7)
Sexual risk factors			
Years in sex work, median (Q1–Q3)	4 (1–8)	4 (1–8)	4 (1–8)
No. of occasional clients in the past week, median (Q1–Q3)	5 (2–14)	3 (1–10)	4 (2–12)
No. of repeat clients in the past week, median (Q1–Q3)	2 (1–5)	2 (1–5)	2 (1–5)
Has regular partner(s), n (%)	208 (58.9)	192 (59.1)	400 (59.0)
Used condom with occasional clients in the past week, n (%)			
Never/sometimes	9 (2.5)	7 (2.2)	16 (2.4)
Every/most times	327 (92.6)	291 (89.5)	618 (91.2)
Not applicable	17 (4.8)	27 (8.3)	44 (6.5)
Used condom with repeat clients in the past week, n (%)			
Never/sometimes	30 (8.5)	31 (9.5)	61 (9.0)
Every/most times	277 (78.5)	240 (73.8)	517 (76.3)
Not applicable	46 (13.0)	54 (16.6)	100 (14.7)
Used condom with intimate partner in the past week, n (%)			
Never/sometimes	175 (49.6)	156 (48.0)	331 (48.8)
Every/most times	33 (9.3)	36 (11.1)	69 (10.2)
Not applicable	145 (41.1)	133 (40.9)	278 (41.0)
Chances of getting HIV, n (%)			
No chances at all	94 (26.6)	67 (20.6)	161 (23.7)
Low	202 (57.2)	227 (69.8)	429 (63.3)
Moderate/high	49 (13.9)	29 (8.9)	78 (11.5)
Do not know	8 (2.3)	2 (0.6)	10 (1.5)
HIV tested in the past year, n (%)	295 (83.6)	295 (90.8)	590 (87.0)
Tested or treated for STIs in the past 3 months, n (%)	103 (29.2)	160 (49.2)	263 (38.8)
Other risk factors			
Uses alcohol, n (%)	220 (62.3)	199 (61.2)	419 (61.8)
Regularly consumes alcohol before sex, n (%)	79 (22.4)	76 (23.4)	155 (22.9)
Used drugs in the past 3 months, n (%)	7 (2.0)	5 (1.5)	12 (1.8)
Physically or sexually abused in the past 3 months, n (%)	42 (11.9)	30 (9.2)	72 (10.6)

PrEP: pre-exposure prophylaxis; STI: sexually transmitted infection.

### Retention and adherence

Of the 79 women (11%) who did not complete all scheduled visits, four died of reasons unrelated to PrEP (one death by suicide, two deaths by homicide, and one death due to cardiac arrest). The remaining 75 dropped out of the study (15-month retention rate of 89%), and all but one of these did so early on during the first 6 months of follow-up (Table 3). The most common reasons for dropping out were as follows: no longer doing sex work (35%), PrEP side effects (19%, see below), and lack of interest (16%). Only four women reported lack of partner support as a cause of dropping out.

**Table 3.** Measures of retention and adherence.

	Enrollment	1 month	3 months	6 months	9 months	12 months	15 months	Total
Total, n (%)	678	544	649	628	599	600	599	
Last visit, n (%)	7 (1)	8 (1)	30 (5)	29 (5)	4 (1)	1 (0)	0 (0)	
Died, n (%)	0 (0)	0 (0)	1 (0.2)	1(0.2)	2 (0.3)	0 (0)	0 (0)	4 (0.6)
Dropped out, n (%)	7 (1)	8 (1)	29 (4)	27 (4)	4 (1)	0 (0)	0 (0)	75 (11)
Reason for drop out								
Not working as FSW	2 (29)	2 (25)	12 (41)	9 (33)	1 (33)	0	0	26 (35)
Side effects	3 (43)	2 (25)	5 (17)	4 (15)	0 (0)	0	0	14 (19)
Not interested	0 (0)	1 (12)	4 (14)	6 (22)	0 (0)	0	0	11 (15)
Other illness	0 (0)	0 (0)	2 (7)	1 (4)	1 (33)	0	0	4 (5)
Daily PrEP inconvenient	1 (14)	1 (12)	2 (7)	0 (0)	0 (0)	0	0	4 (5)
Partner not Supportive	0 (0)	1 (12)	1 (3)	2 (7)	0 (0)	0	0	4 (5)
In police custody	0 (0)	0 (0)	0 (0)	1 (4)	1 (33)	0	0	2 (3)
Embarrassed	0 (0)	0 (0)	2 (7)	0 (0)	0 (0)	0	0	2 (3)
No reason	1 (14)	1 (12)	1 (3)	4 (15)	0 (0)	0	0	7 (9)
No. of pills taken last week								
Overall, median (Q1–Q3)		7 (6–7)	7 (6–7)	7 (6–7)	7 (6–7)	7 (6–7)	7 (6–7)	
7, n (%)		386 (71)	470 (72)	460 (73)	444 (74)	424 (71)	448 (75)	
≥4, n (%)		510 (94)	585 (90)	551 (88)	545 (91)	537 (90)	528 (88)	
0, n (%)		13 (2)	32 (5)	43 (7)	25 (4)	23 (4)	39 (7)	
Took 1 pill every day last month, n (%)		320 (59)	289 (45)	269 (43)	249 (42)	145 (24)	N/A	
Missed ≥ 7 days since last visit, n (%)		63 (12)	116 (18)	123 (20)	118 (20)	119 (20)	129 (22)	
Longest stretch (days) of nonadherence, median (Q1–Q3)		1 (0–3)	2 (0–5)	2 (0–5)	2 (0–5)	3 (0–6)	3 (0–6)	
Plasma tenofovir								
Tested, n (%)		0 (0)	30 (5)	29 (5)	44 (7)	53 (9)	20 (3)	
Levels, ng/mL, median (Q1–Q3)		NA	16 (2–49)	31 (4–48)	30 (3–68)	48 (15–83)	65 (31–173)	
Not detectable, n (%)		NA	3 (10)	3 (10)	5 (11)	2 (4)	3 (15)	
≥10 ng/mL, n (%)		NA	15 (50)	18 (62)	28 (64)	41 (77)	17 (85)	
≥40 ng/mL, n (%)		NA	13 (43)	10 (34)	20 (45)	29 (55)	13 (65)	
Self-assessed ability to adhere (past month), n (%)								
Poor		30 (6)	23 (4)	39 (6)	23 (4)	17 (3)	16 (3)	
Good		198 (36)	279 (43)	253 (40)	299 (50)	288 (48)	316 (53)	
Excellent		316 (58)	347 (53)	336 (54)	277 (46)	295 (49)	267 (45)	

FSWs: female sex workers; PrEP: pre-exposure prophylaxis.

The percentage of women who reported taking at least four pills in the previous week varied over the course of the study, ranging from 94% at month 1, 88% at 6 months, 90% at 12 months to 88% at the last follow-up visit at 15 months (Table 3). The percentage of women who reported taking the recommended daily pill was also stable, at about 73% over the course of the study. However, an average of 20% of women reported not taking their pills for at least 7 days in a row at one point since the last visit, and the median length of the longest stretch of nonadherence increased from 1 day at the 1-month visit to 3 days at the final visit.

Starting at the 3-month visit, women had plasma tenofovir levels measured (Table 3), with the median (Q1–Q3) plasma levels rising from 16 (2–49) ng/mL among those tested at the 3-month visit to 65 (31–173) ng/mL at the 15-month visit. Over the duration of the study, the percentage of women tested whose levels reached ≥10 ng/mL went from 50% at 3 months, 62% at 6 months, 77% at 12 months to 85% at the 15-month visit. Similarly, the percentage of women whose plasma TDF levels reached ≥40 ng/mL went from 43% at 3 months, 34% at 6 month, 55% at 12 month to 65% at the 15-month visit (Table 3).

### Safety

There were no HIV seroconversions observed, and no evidence of a significant change in sexual behavior over the course of the trial (Table 4). The number of occasional and repeat clients and the percentage of women consistently using condoms with such clients stayed constant during follow-up. Furthermore, the percentage of women needing treatment for STIs steadily declined from 39% at enrollment to 13% by the 15-month visit. By the end of the study, a small proportion of women (3%) believed that there was a “moderate to high chance” of becoming infected with HIV compared to 11.5% at enrollment. There were no significant changes in alcohol or illicit drug use or in the risk of violence.

**Table 4.** Measures of risk compensation and safety.

	Enrollment	1 month	3 months	6 months	9 months	12 months	15 months
Sexual health							
No. of occasional clients in the past week, median (Q1–Q3)	4 (2–12)	4 (3–6)	5 (3–7)	4 (3–6)	4 (3–6)	4 (3–6)	3 (2–5)
No. of repeat clients in the past week, median (Q1–Q3)	2 (1–5)	2 (1–4)	2 (1–5)	2 (1–4)	2 (1–3)	2 (1–4)	2 (1–3)
Used condom with occasional clients in the past week, n (%)							
Never/sometimes	16 (2.4)	6 (1.1)	14 (2.2)	9 (1.4)	5 (0.8)	5 (0.8)	3 (0.5)
Every/most times	618 (91.2)	530 (97.4)	626 (96.5)	596 (94.9)	576 (96.2)	574 (95.7)	596 (99.5)
Not applicable	44 (6.5)	8 (1.5)	9 (1.4)	23 (3.7)	18 (3.0)	21 (3.5)	0 (0.0)
Used condom with repeat clients in the past week, n (%)							
Never/sometimes	61 (9.0)	15 (2.8)	14 (2.2)	19 (3.0)	9 (1.5)	14 (2.3)	3 (0.5)
Every/most times	517 (76.3)	521 (95.8)	623 (96.0)	583 (92.8)	559 (93.3)	565 (94.2)	591 (98.7)
Not applicable	100 (14.7)	8 (1.5)	12 (1.8)	26 (4.1)	31 (5.2)	21 (3.5)	5 (0.8)
Used condom with partner in the past week, n (%)							
Never/sometimes	331 (48.8)	283 (52.0)	352 (54.2)	362 (57.6)	339 (56.6)	171 (28.5)	355 (59.3)
Every/most times	69 (10.2)	51 (9.4)	43 (6.6)	35 (5.6)	48 (8.0)	255 (42.5)	56 (9.3)
Not applicable	278 (41.0)	210 (38.6)	254 (39.1)	231 (36.8)	212 (35.4)	174 (29.0)	188 (31.4)
Tested or treated for STIs in the past 3 months, n (%)	263 (38.8)	171 (31.4)	158 (24.3)	109 (17.4)	85 (14.2)	90 (15.0)	76 (12.7)
HIV risk perception, n (%)							
No chances at all	161 (23.7)	290 (53.3)	367 (56.5)	398 (63.4)	407 (67.9)	434 (72.3)	426 (71.1)
Low	429 (63.3)	221 (40.6)	248 (38.2)	206 (32.8)	172 (28.7)	146 (24.3)	156 (26.0)
Moderate/high	78 (11.5)	33 (6.1)	32 (4.9)	24 (3.8)	20 (3.3)	20 (3.3)	16 (2.7)
Do not know	10 (1.5)	0 (0.0)	2 (0.3)	0 (0.0)	0 (0.0)	0 (0.0)	1 (0.2)
Other risk factors							
Uses alcohol, n (%)	419 (61.8)	325 (59.7)	386 (59.5)	379 (60.4)	354 (59.1)	378 (63.0)	380 (63.4)
Used drugs in the past 3 months, n (%)	12 (1.8)	3 (0.6)	9 (1.4)	6 (1.0)	1 (0.2)	7 (1.2)	10 (1.7)
Physically or sexually abused in the past 3 months, n (%)	72 (10.6)	31 (5.7)	27 (4.2)	16 (2.5)	18 (3.0)	15 (2.5)	16 (2.7)
Adverse events (since last visit)							
Any, n (%)	NA	167 (30.7)	88 (13.6)	45 (7.2)	18 (3.0)	0 (0.0)	0 (0.0)
Nausea/vomiting, n (%)	NA	131 (24.1)	64 (9.9)	30 (4.8)	10 (1.7)	0 (0.0)	0 (0.0)
Dizziness, n (%)	NA	42 (7.7)	27 (4.2)	16 (2.5)	5 (0.8)	0 (0.0)	0 (0.0)
Fatigue, n (%)	NA	33 (6.1)	21 (3.2)	9 (1.4)	5 (0.8)	0 (0.0)	0 (0.0)
Headache, n (%)	NA	18 (3.3)	12 (1.8)	10 (1.6)	3 (0.5)	0 (0.0)	0 (0.0)
Rash, n (%)	NA	8 (1.5)	2 (0.3)	2 (0.3)	0 (0.0)	0 (0.0)	0 (0.0)
Other, n (%)	NA	7 (1.3)	8 (1.2)	3 (0.5)	1 (0.2)	0 (0.0)	0 (0.0)

STIs: sexually transmitted infections.

As noted above, there were 4 deaths during the course of the study, all unrelated to PrEP. About 31% of women reported one or more adverse effects during the first month of use, most commonly nausea/vomiting (24%), dizziness (8%), and fatigue (6%). By the 9-month visit, only 3% reported such effects, and by the 12-month visit, there were no reports of any adverse effects. Fourteen women dropped out of the study because of minor adverse events, all within the first 6 months of the study.

## Discussion

Our study demonstrated very high levels of retention and adherence to PrEP among participating FSWs. Whereas PrEP has consistently been shown to be effective at preventing HIV infection in RCTs of men who have sex with men (MSM),^6,24,25^ results among women have been less encouraging: two major clinical trials (VOICE^26^ and FEM-PrEP^27^) conducted in South Africa found no evidence of reduced risk among heterosexual women.^28^ This was attributed to poor adherence (<30% of participants having detectable drug levels) in both studies. Smaller studies (TDF2-Botswana^9^ and Bangkok-TDF^29^) with better adherence (>75%) did show substantial effectiveness (RR 0.35, 95%CI 0.22–0.54),^21^ and a recent demonstration trial from Senegal demonstrated high levels of retention and no incident HIV infections.^30^ In our trial, most tested women had TDF levels suggestive of consistent TDF-FTC use, particularly among those tested in later visits. Importantly, there were no HIV seroconversions detected.

In addition to the good adherence noted in this population, there was a very low study dropout rate (11%), with only a few women dropping out because of lack of interest, inconvenience, stopping sex work, or partner opposition. These high levels of retention and adherence are in contrast to other PrEP studies which have been implemented among FSWs and high-risk women.^9,26,31^ We believe that this was likely due in large part to the community outreach and support provided by DMSC, a long-standing community-owned HIV prevention organization. In several other contexts, these levels of retention and adherence have not been demonstrated. For example, in a PrEP trial conducted in Benin, adherence among FSWs at the final visit was only 43%.^19^ The authors suggest that the lack of retention and adherence in the Benin study was due to the high levels of mobility among FSWs. The treatment and prevention for female sex workers study in South Africa also had very high rates of loss to follow-up, with only 22% of FSWs completing a 12-month visit. However, among those participants who were seen in follow-up, self-reported adherence was high (70–85%).^32^ A key population-led model for the delivery of PrEP for MSM and transgender women in Thailand demonstrated high levels of self-reported adherence, but retention remained an issue, with only 44% of participants completing the final 12-month visit.^33^ A similar study to ours was conducted in South India, led by a long standing community-owned HIV prevention organization, and it also demonstrated both high levels of adherence as well as retention.^13^

There was no evidence of significant changes in self-reported sexual behavior or other measured risk factors, such as alcohol and drug abuse. While these reports could have been distorted by lack of blinding or social desirability bias, the proportion of women treated for STIs, a more objective marker for sexual risk behavior, declined significantly over time, supporting the notion that PrEP use was not associated with increased risk in sexual behavior. Reported condom use was consistently high with clients and consistently low with intimate partners, as has been seen in other HIV prevention programs in India and globally,^2,19,32,34^ and this did not change over the course of the study. While some studies have seen evidence of risk compensation with the use of PrEP,^12,35,36^ this has not been consistently seen across all trials or all populations.^37,38^

Participants in our study were given the choice between two PrEP delivery options: weekly clinic pickup or home delivery by a peer educator every other day. Approximately half of participants chose clinic pickup and half chose home delivery, and no significant differences were seen between PrEP users who chose one delivery method over the other. Despite substantial evidence on the effectiveness of PrEP, there has been slow uptake globally. Delivery method has been noted as a potential barrier to PrEP uptake, and the need for innovative provision strategies to increase uptake has been identified.^39–41^

There were no severe adverse events observed in this trial, and only a small percentage of participants dropped out because of adverse events. Similar to what was observed in the RCTs evaluating PrEP, mild gastrointestinal side effects were noted early on after initiation of PrEP,^21,42,43^ affecting about 25% of participants. Generally, these were mild and self-limited among participants, with only 14 (2.0% of enrolled participants) withdrawing from the study as a result.

### Limitations

We did not measure PrEP acceptability in the general FSW community, but of note, there has been a high demand in that community for access to PrEP. After completion of the trial, 78% of former participants indicated that they were prepared to pay for PrEP in order to continue using it, and 63% continued for at least 3 months following completion of the study. Another limitation of the study is that participants were self-selected, so may not be representative of the larger source population. However, our study population did share similar characteristics to the larger sex worker population engaged in DMSC programs (the average age of DMSC program registrants is 28 years, the same as in our study, and the average reported number of clients in the past week is three vs four in our study). Adherence was measured by both self-report and TDF levels. However, possibly due to social desirability bias, the self-reported adherence rates were higher than adherence rates measured through blood drug levels. Our study did not examine cost-effectiveness, but research by Vickerman et al. suggests that PrEP is cost-effective for FSWs.^44^

### Conclusions

This trial demonstrated that the integration of PrEP into a community-based HIV prevention program for FSWs can be feasible and effective. As shown, PrEP was safe and well tolerated, and women adhered very well to the regimen. We strongly believe that the community-based support and outreach inherent in the DMSC program was integral to the success of the PrEP program. As other PrEP studies have shown, adherence to PrEP is essential in order for it to be effective, and when adherence is ensured, PrEP is a highly effective HIV preventive intervention.^31^ This study has generated important information for HIV programs for FSWs, and the results can be used to inform HIV prevention policies in India and globally.
